# Differentiated service delivery strategies to optimize modern contraceptive uptake among adolescents with HIV in Northern Uganda

**DOI:** 10.4314/ahs.v25i1.10

**Published:** 2025-03

**Authors:** Harriet Ajilong, Felix Bongomin, Pebalo F Pebolo, James H Obol

**Affiliations:** 1 Department of Pediatrics and Child Health, Faculty of Medicine, Gulu University, Gulu, Uganda; 2 Department of Medical Microbiology and Immunology, Faculty of Medicine, Gulu University, Gulu, Uganda; 3 Department of Reproductive Health, Faculty of Medicine, Gulu University, Gulu, Uganda; 4 Department of Public Health, Faculty of Medicine, Gulu University, Gulu, Uganda

**Keywords:** Modern contraceptive methods, HIV-positive adolescents, Differentiated Service Delivery Strategies

## Abstract

**Background:**

In sub-Saharan Africa, contraceptive use among adolescents in 2014 ranged from 21–42% with an unmet need of 53-64%. Therefore, client-centered strategies like the differentiated service delivery (DSD) model should be explored to improve contraceptive use among adolescents in northern Uganda.

**Methods:**

An exploratory mixed-methods study was conducted in HIV positive adolescents 12-19 years at Gulu hospital. Structured questionnaires were used to obtain information on contraceptive use, and the most convenient mode of delivery. Selected key informants identified specific delivery strategies potentially associated with higher contraceptive uptake.

**Results:**

Of the 193 respondents who participated in the study, 108(56%) were females with a mean age of 15 years. Approximately 20% of the respondents were sexually active. Ever use of modern contraceptives was low at 16% because only a small percentage(approximately 20%) were sexually active. Most participants, 54/193 (40%) preferred accessing contraceptives from the adolescent clinic from health workers and peer supporters. Factors associated with increased contraceptive use were tertiary levl education and employment. From qualitative analysis participants recommended separation of adolescent services, peer support and community outreaches to improve uptake.

**Conclusion:**

The contraceptive prevalence rate among HIV-positive adolescents is still low. Contraceptive use is higher among the educated or employed. Services should be provided at adolescent-friendly clinics by trained health workers and peers during school holidays.

## Introduction

Despite the documented benefits of contraception, World Health Organization (WHO) estimates the global unmet need for contraceptives among adolescents aged 15–19 years as approximate-23 million and that more than 50% of pregnancies among this age group are unintended^1^. In sub-Saharan Africa (SSA), contraceptive use among adolescents aged 15–19 years in 2014 ranged from 21–42% with an unmet need of 53–64% among those who were unmarried compared to 16–62% among their married counterparts^2^–^4^.

In Uganda, the contraceptive prevalence among adolescents ranges from 10-35% with variations among those in rural and urban settings^5^. Studies have shown that adolescents in Uganda start having sex at an early age with the median age at first intercourse at 16 years^6^.

Most of the sexual encounters in this age group are unprotected thereby exposing these adolescents to unwanted pregnancies and sexually transmitted infections including HIV^7^,^8^. According to the latest Uganda Demographic Health Survey, about 24.8% of girls aged 15-19 had already begun child-bearing^9^. Furthermore in 2020, adolescents accounted for 14% of institutional maternal deaths and yet only 7% were facility deliveries^10^. Complications of pregnancy and childbirth are worse among adolescents and are a leading cause of death among women of reproductive age^11^,^12^. Uganda's national policy on adolescent health allows sexually active adolescents to access contraceptives without the consent of their parents or guardians. However, with only 26% of facilities having adequately trained staff in adolescent and youth friendly (AYFS) services, too few adolescents can access responsive care^13^. Other barriers to care include financial constraints of accessing contraceptives, poor contraceptive knowledge, individual myths, and misconceptions among others^3^,^14^. A study that investigated the prevalence and factors associated with modern contraceptive utilization among Ugandan female adolescents aged 15–19 years showed that utilization was at 9.4%^15^. This is very low and negatively affects Uganda's progress in achieving sustainable development goal (SDG) 3 aimed at ensuring universal access to sexual and reproductive healthcare services, including family planning by 2030^16^. The low contraceptive use and high adolescent fertility rates imply that a lot must be done to increase access and utilization in this population to reduce unintended pregnancies and associated mortality and morbidity. Therefore, innovative strategies like differentiated service delivery (DSD) which are client-centered and adolescent friendly should be explored to bring services closer to the users and reduce unnecessary burdens on the health system^17^. These models include(i) facility-based individual models such as fast-track, where individuals collect their commodity refills at the health facility without queuing or seeing a clinician (ii) Out-of-facility individual models, where individuals collect their drug refills from fixed community distribution points or community pharmacies (ii) Healthcare worker-managed groups in facilities or communities, such as teen clubs where a group of 10-30 clients book to collect their drugs at the same time as a group, usually within a health facility (iv) Client-managed groups where groups of 4-12 clients in a given community nominate a group leader who collects medication for the rest of the group.

The DSD model has been scaled up in many countries to improve uptake and adherence to antiretroviral therapy among HIV-infected populations, however, this strategy has not been explored to improve the uptake of modern family planning methods among adolescents^18^,^19^. For example, in Malawi and Tnzania, contraceptive use was negligible among women living with HIV however when contraceptive services were integrated into the facility fast-track model, uptake improved by more than 50%^20^,^21^. Another qualitative study conducted in Malawi among adolescents aged 15-19 years revealed that participants preferred privately owned pharmacies and community-based spaces as access points for their contraceptives. Majority of the respondents were reluctant to access these commodities from health facilities for fear of discloure of this information to their family members^22^. Uganda's health sector strategic plan for 2020-2025 addresses its policy on the procurement and distribution of contraception to all males and females but especially focuses on adolescents^23^. However, there is still an existing gap between the policy and utilization of these services in this age group^24^,^25^. Therefore, we aimed to evaluate the various differentiated service delivery strategies that are adolescent-friendly and client-centered that can improve modern contraceptives uptake among HIV-positive adolescents in Northern Uganda

## Methods

### Study design and setting

The study which was conducted at the adolescent HIV clinic at Gulu Regional Referral Hospital (GRRH) from April to June 2023 was a mixture of a cross-sectional quantitative study design while the qualitative component was based on grounded theory. A mixed methods study with both quantitative and qualitative components was conducted at the adolescent HIV clinic at Gulu Regional Referral Hospital (GRRH) from April to June 2023. Gulu RRH is in the northern Ugandan city of Gulu, approximately 340 kilometers north of Kampala, Uganda's capital city. The adolescent HIV/ART clinic is located within the hospital premises. It serves about 500 active HIV-positive adolescents' majority of who come from the city center and its surroundings. Services offered include HIV testing and counseling, testing of sexually transmitted infections (STIs), and treatment of comorbidities. Contraceptive services are accessed mainly by adults. Adolescents attending the antiretroviral (ART) clinic were specifically chosen for this study because they are a vulnerable group with inadequate access to modern contraceptives.

### Sample size

Kish Leslie 1965 was used to calculate the sample size as follows; using 20% as the proportion of adolescents (15-19 years) who used modern contraceptives in the UDHS survey 2016, a precision of 0.005 and assuming 95% level of confidence, the sample size estimation was 245 participants. However When we adjusted for 15% non-responsiveness, the final sample size was 205 but because majority of the participants were school going, we were able to recruit 193 participants from the adolescent HIV clinic.

### Participant recruitment

HIV positive adolescents aged 12-19 years attending the HIV/ART clinic in Gulu RRH were were consecutively conveniently enrolled into the study after obtaining informed consent. For the qualitative component, we drew a purposive sample of 20 participants based on their age, expert knowledge, and responsibility. The participants included adolescents aged 12–19 years and health care workers at the ART clinic. The adolescents were: still in school, out of school, married or unmarried, and willing to participate in the study with parental consent.

### Data collection

To examine the contraceptive behavior of the study participants, structured questionnaires containing socio-demographics, knowledge on contraception, exposure to mass media, sexual behavior and lifetime use of any modern contraceptive were administered to the participants by trained research assistants at the adolescent HIV/ART clinic.

The question on improving access/uptake was answered through in-depth interviews with selected adolescents and health workers by experts in qualitative research using interview guides developed from study objectives and the International Aids Society (IAS) adapted conceptual framework. Participants were asked to provide the most convenient mode of delivery of contraceptive commodities: where, when and by whom. The interviews were conducted in a private room at the clinic to ensure privacy and confidentiality. The responses during each face-to-face interview were digitally recorded, transcribed, and translated into the local language (Acholi) for those who do not understandnglish. All audios were identified by a number to ensure anonymity and data were stored in a computer protected by a password.

### Data analysis

Quantitative data was analyzed using Stata Version 15.0. Continuous variables were summarized continuous variables as mean (standard deviation) and median (interquartile range) as appropriate and categorical variables as frequencies and percentages. We used modified Poisson regression analysis with robust standard errors to perform multivariable analysis. Variables with a p < 0.2 at the bivariable level, those known to be associated with modern contraceptive use from literature and those considered plausible although not significant, were entered into the multivariable model to determine the independent factors associated with modern contraceptive use.

For the qualitative component, we used thematic content analysis to analyze the data using Atlas ti.9. The analysis followed a five-step process. First, we read through the transcripts and became familiar with the data. Secondly, we organized data in a meaningful way and generated the initial codes. Once the data had been sufficiently coded and saturation reached, themes were identified. Themes were then reviewed and modified, and all data relevant to each theme were gathered. Finally, we defined the themes to identify the real meaning of each theme that had been developed.

### Ethical approval

Ethical clearance to conduct the study was obtained from Gulu University Research Regulatory Ethics Committee (GUREC-approval number 2022-407) and the Uganda National Council of Science and Technology (UNCST-HS4819ES). Permission to recruit participants from the adolescent clinic was obtained from authorities at Gulu RRH. Participation in the study was voluntary and written informed consent was obtained from all study participants before the interviews. Written parental Parental consent and adolescent assent were obtained from all participants aged below 18 years whereas those aged above 18 years provided their own consent per regulations that govern research in Uganda.

## Results

### Participant characteristics

A total of 193 participants were enrolled. More than a half 53.4% (n=103) were aged between 15 and 19 years. The mean age± standard deviation for all participants was 15.2 ±2.5 years. Majority 66.3% (n=128) had attained primary level of education and almost 95.3% (n=184) were unemployed. The socio-demographic characteristics of the adolescents are summarized in Table 1 below.

**Table 1:** Sociodemographic characteristics of HIV positive adolescents aged 12-19 years at Gulu RRH

Characteristic (n=193)	Frequency (%)
**Sex of the participant**	
Female	108(56.0)
Male	85(44.0)
**Age of the participant in years, Mean ±SD: 15.2 ±2.5** years	
12 to 14	90 (46.6)
15 to 19	103(53.4)
**Religion**	
Catholic	109(56.5)
Evangelical	10(5.2)
Others	8(4.2)
Protestant	66(34.2)
**Highest education level attained**	
None	12(6.2)
Primary level	128(66.3)
Secondary level	45(23.3)
Tertiary level	8 (4.2)
**Employment status**	
Employed	9(4.7)
Not employed	184(95.3)
**Marital status**	
Married/Cohabiting	4(2.1)
Never married	188(97.4)
Separated/Divorced	1(0.5)
**Distance to ART Clinic**	
≥5km (More than five-kilometer distance to the ART clinic)	69 (35.8)
≤5km (Less or equal to five-kilometer distance to the ART clinic)	124 (64.25)

### Sexual and reproductive characteristics of the participants

Less than a quarter 19.7% had ever had sex and majority reported that it was consensual sex. More than two thirds first had sex when aged between 15 and 19 years. Among the ever married (n=5), the mean age at getting married was 17.2± 1.8 years and among those who have ever become pregnant, the mean age at first pregnancy was 17± 1.4 years. The sexual and reproductive characteristics of the participants are summarized in Table 2 below.

**Table 2:** Sexual and reproductive characteristics of HIV positive adolescents aged 12-19 years at Gulu RRH

Characteristic (n=193)	Frequency (%)/Mean ±SD
**Ever had sex**	
No	155(80.3)
Yes	38(19.7)
**Consensual or forced sex (n=38)**	
Consensual	37(97.4)
Forced (rape/defilement)	1(2.6)
**Age at first sex (n=33)**	
9 to 14	4 (10.512.1)
15 to 19	3429(89.587.9)
**Age when got married (n=5)**	17.2± 1.8
**Age at first pregnancy (n=7)**	17± 1.4
**Total number of pregnancies (n=6)**	1.2± 0.4

### Contraceptive behavior and preferences for the participants

Majority 71.0% (n=137) had ever heard about family planning. Half of the participants reported schoolteachers and matrons as the source of information on family planning. A total of 22 out 193 (16.1%) had ever used modern contraceptives and majority 86.4% (n=19) reported use of condoms. Regarding the convenient place to get contraceptives, more than a third 39.4% (n=54) reported the Adolescent clinic/ART clinic as the most convenient place and more than two thirds 68.9% (n=133) reported a health worker as the convenient person to offer contraceptive services. Further, more than half 55.5% (n=76) of the adolescents reported school holidays as the most convenient time to receive contraceptives. The contraceptive behavior of the study participants is summarized in Table 3 below.

**Table 3:** Contraceptive behavior and preferences of HIV positive adolescents aged 12-19 years at Gulu RRH

Characteristic (n=193)	Frequency (%)
**Ever heard about family planning**	
No	56(29.0)
Yes	137(71.0)
**Source of information**	
One on one from health worker	10(7.3)
Health education talks	35(25.6)
Radio/newspaper	51(37.2)
Social media	7(5.1)
School teachers/matron	69(50.4)
Peer/friend	35(25.6)
Other, specify	26(18.9)
**Ever used any modern contraceptive method**	
No	115(83.9)
Yes	22(16.1)
**Modern contraceptive used**	
Condoms	2019(90.986.4)
Pills	1(4.54.6)
Implants	1(4.54.6)
**Source of contraceptives**	
Adolescent HIV/ART clinic	46(24.0)
Community Youth Center	9(4.7)
Community health worker/VHT	3(1.6)
Community pharmacy/drug shop	4(2.1)
Nearby health facility	39(20.3)
Don't know	8584(44.043.8)
School	7(3.7)
**Most convenient place to get contraceptives**	
Adolescent HIV/ART clinic	54(39.4)
Community Youth Center	6(4.4)
Community health worker/VHT	2(1.5)
Community pharmacy/drug shop	1(0.7)
Nearby health facility	32(23.4)
Don't know	40(29.2)
School	2(1.5)
**Most convenient person to offer contraceptives**	
Community health worker/VHT	3(1.6)
Health worker	133(68.9)
Other, specify*	13(6.7)
Parent/guardian	18(9.3)
Peer counselor	14(7.3)
Peers/expert clients	9(4.7)
Teacher	3(1.6)
Other	13(6.7)
**Frequency of receiving contraceptive supplies?**	
Every 3 months	28(20.4)
Every 6 months	31(22.6)
Every week	4(2.9)
Other, specify	74(54.0)
**Most convenient time to receive contraceptive supplies**	
After working hours	4(2.9)
During working hours	2(1.5)
On weekends/public holidays	15(11.0)
Other, specify	34(24.8)
School holidays	76(55.5)
School times	6(4.4)
Other	34(24.8)

### Factors associated with modern contraceptive use among HIV-positive adolescents in Gulu district

Adolescents with a tertiary level of education had 5.5 times the odds of using modern contraceptives as compared to those with no education. a secondary and primary level of education. [APR: 5.55; 95% CI: (1.81-17.06)]. While adolescents who were unemployed had 77% less odds of using modern contraceptives as compared to those were employed. [APR: 0.23; 95% CI: (0.09-0.56), table 4

**Table 4:** Factors associated with modern contraceptive use among HIV-positive adolescents aged 12-19 years at Gulu RRH

Variable	Crude Prevalence Ratio	95% CI	p-value	Adjusted Prevalence Ratio		95% CI p-value
**Sex of the participant**						
Female	1			1		
Male	2.25	(1.01-5.01)	0.048	1.97	(0.77-5.01)	0.155
**Age of the participant in years**						
12 to 14	1			1		
15 to 19	9.94	(1.37-72.03)	0.023	7.08	(0.94-53.11)	0.057
**Religion**						
**Catholic**	1			1		
Protestant	0.84	(0.36-1.94)	0.683	0.69	(0.31-1.50)	0.346
Others	0.51	(0.07-3.55)	0.499	0.25	(0.04-1.38)	0.111
**Highest education level attained**						
None	1			1		
Primary level	0.3	(0.08-1.13)	0.075	1.28	(0.47-3.50)	0.637
Secondary level	0.41	(0.11-1.59)	0.197	1.07	(0.32-3.62)	0.91
Tertiary level	2.25	(0.67-7.50)	0.187	5.55	(1.81-17.06)	**0.003**
**Employment status**						
Employed	1			1		
Not employed	0.24	(0.11-0.53)	0	0.23	(0.09-0.56)	**0.001**
**Distance to ART Clinic**						
≥5km (More than five-kilometer distance to facility)	1			1		
≤5km (Less or equal to five-kilometer distance to facility)	0.86	(0.39-1.90)	0.701	0.76	(0.35-1.67)	0.498

### Proposed strategies for optimizing uptake of contraceptives among adolescents in Gulu district from key informant interviews

#### Health system-based delivery strategies

The health workers interviewed of whom the majority were nurses reported that contraceptive supplies particularly condoms were readily available at the adolescent clinic, and they were willing to distribute them to the adolescents.

“We have condoms, we usually supply, and we place some in the safety box for our clients” (Health worker _1)

“Yes, I am comfortable giving adolescents contraceptives because this will reduce the rate of teenage pregnancy that is occurring so widely”. (Health worker_4)

Contraceptive service delivery should be strengthened by ensuring that facilities have a conducive designated space where adolescents can privately access all the services needed. From the interviews, several participants recommended separation of adolescent from adult services to improve demand and access for contraceptives by the younger population.

“Because these adolescents fear that whenever they are getting these services, maybe somebody is seeing them, they fear stigma, so if possible if they can provide a room specifically for adolescents to access family planning” (Health worker_8)

“I think, the clinic for adolescents should be different…you cannot mix an adolescent with adults. In the hospital they should have a corner for youth or adolescents” (17-year-old adolescent_1)

Furthermore, the health system should invest in training young people to provide integrated reproductive health services. The respondents felt that to promote delivery of contraceptive services, the young people and adolescent peer supporters (YAPS) should be engaged since the adolescents can freely interact with them.

“The YAPS should be the ones providing these services…because these adolescents are free with the YAPS because they are their age mates, and they can talk their language.” (Health worker_9)

“I think that one is safer with a fellow adolescent providing the service because you have many questions to ask, and you are also free to interact. It is easy to approach and tell your age mate your issues” (19-year-old adolescent_4)

#### Community-based delivery strategies

Some respondents suggested that contraceptive services should be taken nearer to the users through community outreaches. They also felt that community health workers should be trained to provide these services to make them more accessible.

“And then during the outreaches, we take the services to the outreach point. Its nearer to the community and so easy to get”. (Health worker_6)

“I think it would be better if they are uncomfortable getting it from here, maybe we put the contraceptives in centers where they can access it without coming to the hospital; okay, like decentralizing the service points…. maybe they put in the boda centers there or in the markets”. (Health worker_4)

“Some people will not have the time to move long distance to get the service. The family planning methods should be given to the community health worker at home…because that will be nearer to us” (18-year-old adolescent _5)

## Discussion

This study outlines differentiated delivery strategies to optimize uptake of modern contraceptives among HIV -positive adolescents 12-19 years. The study further highlights the contraceptive prevalence in this population and factors associated with contraceptive use. From our findings, 1 in 5 adolescents reported being sexually active and the mean age at first pregnancy was 17 years. Majority had their sexual debut during late adolescence at 15-19 years. These findings are similar to the 2016 Uganda Demographic Health Survey (DHS) survey where almost 25% of adolescents aged 15-19 in Uganda had reportedly begun childbearing.

### Modern contraceptive prevalence among adolescents 12-19 years

Modern contraceptive use in this population was lower (16%) than that in the UDHS 2016 survey among older adolescents^9^. The lower utilization rate among adolescents could be attributed partly to limited contraceptive knowledge, service provider misconception and attitudes about contraceptive service provision to adolescents, inadequate adolescent-friendly services among others^25^. This prevalence is still low and negatively affects Uganda's progress of achieving sustainable development goal (SDG) 3 target aimed at ensuring universal access to sexual and reproductive health-care services, including family planning by 2030^10^. Condoms were the most popular modern method among the adolescents and a big percentage obtained their contraceptives from the adolescent HIV clinic. Factors associated with contraceptive use were tertiary level of education and employment. Participants who were educated up to tertiary level had 5.5 times the odds of using modern contraceptives as compared to those with a secondary and primary level of education. [APR: 5.55; 95% CI: (1.81-17.06)]. Adolescents who were unemployed had 77% less odds of using modern contraceptives as compared to those were employed. [APR: 0.23; 95% CI: (0.09-0.56)] Education and a higher social economic status obtained through employment increases autonomy and decision-making skills about reproductive health choices^5^. There is a need to design programs to keep HIV-infected adolescents in school to increase their autonomy in reproductive health decision-making. Furthermore, those who are out of school should be engaged in gainful employment so that they can afford these reproductive health services.

### Contraceptive delivery strategies for adolescents

From this study, more than a third 39.4% (n=54) reported the adolescent clinic/ART clinic as the most convenient place and more than two thirds 68.9% (n=133) reported a health worker as the convenient person to provide these services. Further, more than half 55.5% (n=76) of the adolescents reported school holidays as the most convenient time to receive contraceptives.

However, from the qualitative analysis, respondents preferred obtaining their contraceptive supplies from peers and from the community. These findings are consistent with a study in Malawi where adolescents suggested accessing contraceptives from various sources including local drug stores, pharmacies, and hospitals at a health system level and through Youth Centers, clubs, and corners at a community level^22^. Thus, adolescent sexual and reproductive health programs need to consider a wide range of outlets where adolescents can access contraceptives, leverage and integrate contraceptive services with existing health services and make all these access points adolescent-friendly^27^,^28^. Furthermore, it is important to build community support for the provision of contraception to adolescents, build the capacity of non-health service providers including peer supporters and community health workers in modern contraceptive service provision^28^. Convenient service provision times that are responsive to adolescents' needs such as out of school hours and school holidays should be considered to optimize access^28^.

### Strengths and limitations of the study

One strength of this study is that it was a mixed study combining both quantitative and qualitative methods. This helped the study to capture the views of implementers of the strategies such as health care workers and adolescents who are users of the contraceptives. Therefore, the study was able to explore factors that can influence contraceptive uptake and hence the findings can potentially be used to inform further research, policy, and practice.

The study limitation was in its cross-sectional design thereby making it difficult to demonstrate causality. Furthermore more, sexual behavior was reported and hence there could have been potential of recall bias or underreporting since participants may have been embarrassed to reveal information about their sexual behavior.

## Conclusion

The study found that the modern contraceptive prevalence rate among HIV-positive adolescents is still low and yet they are sexually active. Adolescents were more likely to use modern contraceptives if they were more educated or employed. Therefore, in addition to making health facility and community interventions adolescent friendly, wholistic strategies that empower adolescents in reproductive health decision making including keeping them longer in school or empowering them social-economically should be strengthened to improve access and contraceptive use in this population.

## Data Availability

To protect the confidentiality of the participants, ethical restrictions have been imposed on the data in this study. Interested researchers may submit queries related to data access through the corresponding author (harrietajilong@gmail.com)
